# Identification of HPV16's E6 gene in suspected cases of cervical lesions and docking Study of its L1 protein with active components of *Echinacea purpurae*

**DOI:** 10.4314/ahs.v22i1.13

**Published:** 2022-03

**Authors:** Yusuf Lukman, Doro A Bala, Kabir I Malik, Abdulkadir Saidu, Kumurya A Saleh, Bala J Abubakar, Aliyu I Abubakar

**Affiliations:** 1 Microbiology unit, Department of medical laboratory science faculty of allied health science, Bayero University kano, Nigeria, P.M.B 3011, Kano, Nigeria; 2 Virology unit, Department of pathology and Microbiology, Faculty of Veterinary Medicine, Universiti Putra Malaysia, Malaysia, 43400, Serndang, Selangor Darul Ehsan, Malaysia; 3 Federal Medical Centre Katsina, PMB 2121, Katsina, Nigeria

**Keywords:** HPV, Echinacea purpurae, chicoric acid, echinacoside, curcumin

## Abstract

**Background:**

HPV 16 is the primary etiologic agent of cervical cancer and the presence of L1 and E6 oncoproteins are largely responsible for its virulence. It was the objective of this study to identify HPV16 isolates from suspected cases of cervical cancer at Specialist Hospital Sokoto and Sir Yahaya Memorail Hospiatal Birnin Kebbi, Nigeria and also to identify potent HPV16's L1 protein inhibitor using in silico analysis.

**Methods:**

A total of 144 cervical samples consisting of 21 low grade squamous intraepithelial lesion, 6 high grade lesion and 117 negative pap smears were collected. The samples were subjected for molecular detection using PCR targeting E6 gene of the virus. Data generated for the molecular prevalence was statistically analyzed using Chi-square method. AutoDock Vina was used to carry out the molecular docking between 2hr5 and Chicoric acid, curcumin and Echinacoside.

**Results:**

Out of the 144 samples, 24 samples were positive for the PCR representing 16.9% molecular prevalence rate. There is statistically significant association between cyto-diagnoses and presence of HPV16 (P < 0.05). Docking analysis showed that the Chicoric acid components of Echinacea purpurae have strong binding affinity (-8.7 kcal/mol) to the L1 protein of the HPV.

**Conclusion:**

This study provides data on HPV 16 epidemiology in northern Nigeria, and also provides novel evidence for consideration on certain interacting residues, when synthesizing Anti-HPV compounds in the wet lab.

## Introduction

Human papillomavirus (HPV) is one of the most prevalent infections that are transmitted during sexual activities affecting both male and female gender^1^. The HPV genome is approximately 8kb in size, the genome is chromatinized, double stranded DNA which is enclosed in a 55 nm icosahedral capsid^2^. At the onset of HPV infection the virion attaches to heparin sulfate proteoglycans (HSPGs) situated in the cell surface^3^, in order to induce conformational change in L1 and L2 proteins, the viral L1 proteins will engage with HSPGs^4^, then the host cell cyclophillin B facilitates the exposure of N-terminus, which reveals a furin convertase cleavage site^5^. Following the conformational changes, the virion is associated with certain non-HSPG receptors such as tetrasparins, annexin A2, growth factor receptor and integrins, to facilitate viral entry^6^. Although, as an important receptor molecule for HPV infection, annexin A2 is involved in both virus attachment and internalization^7^, after the viral attachment the virion enters the host through the endocytic route^6^. On human keratinocyte exposure, HPV was observed to cause activation of EGFR dependent-Src kinase that results in phosphorylation of annexin A2 Tyr23 and subsequent extracellular translocation to the outer plasma membrane leaflets This led to annexin A2 been mobilise to the cell surface^7^. Human Papillomaviruses disease progression ranges from benign lesions to malignant^8^. The HPV types that are carcinogenic to the mucous membrane belongs to the genus alpha-papillomavirus, and the HPV types (16 and 18) are the major cause of cancer of the cervix^9^. These HPV types shows presence of E6 encoded oncoproteins, which are largely responsible for virulence and pathogenicity. E6 gene binds and inactivate the tumour suppressor gene P53, leading to carcinogenesis^9^.

Echinacea purpurae is one of the world's most common medicinal plant and belongs to the Astracea family^10^. It is widely used in the upper respiratory tract as a chemotherapeutic plant in particular. It is also used in the treatment of cervical cancer and function as an immunomodulators. Although the isolation and structural elucidation of its main compound has been noted by researchers, yet there is no conclusion on its mechanism of action^11^. A potent antiviral photosensitiser was seen in the ethyl acetate and ethanol soluble fractions of the plants stem and leaves^12^. Another molecular docking research in which one of the plant's components, L-chicoric acid was docked against the protein HIV-1 (Human immunodeficiency virus type 1) integrase, showed very good binding modes between the ligand and the viral integrase^13^. Animal studies of different preparations of Echinacea species have also demonstrated low toxicity^11^.

In most parts of northern Nigeria, the prevalence of HPV is unknown, therefore the main objective of this study is to identify HPV16 isolates from suspected cases of cervical lesion at Specialist Hospital (SH) Sokoto and Sir Yahaya Memorial Hospital (SYMH) Birnin Kebbi and also to identify potential inhibitors for HPV16's L1 protein using in silico analysis of echinacoside, curcumin and chicoric acid against the viral protein.

## Materials and methods

### Clinical samples

A total of 144 women with an average age of 33 years (range 20–60 years) participated in this study. The population was recruited from patients attending obstetrics and gynaecology units of SYMH Birnin Kebbi and SH Sokoto, Nigeria.

Cervical smear was collected from each of the participants by an application of standard procedure using sterilized speculum and swab (cyto-brush).14. this study adopted the following cytological classification: Negative; for normal cytology, low grade intraepithelial lesion, high grade intraepithelial lesion and carcinoma in situ; for malignancy.

### Inclusion criteria

Consented patients aged 18 years and above attending attending obstetrics and gynaecology units of the selected hospitals.

### Exclusion criteria

Non-consented patients, pregnant women and participants with vagina bleeding were excluded from the study.

### Polymerase Chain Reaction

DNA extraction

DNA extraction was done using Viral Nucleic Acid Extraction kit II (Geneaid Biotech LTD, Taiwan). Extraction was carried out following the manufacturer's instruction, stated as follows: Two hundred (200) µl of sample was dispensed ınto1.5ml tube, then 400 µl of VB lysis buffer was added into the tube containing the samples, mixture was vortexed, then incubated at room temperature for 10 minutes. Four hundred and fifty (450) µl AD buffer was added into sample lysate and vortexed vigorously, VB column was placed in 2ml collection tube, 600 µl of lysate mixture was added into the VB column and centrifuged at 16,000g for 1 minute, flow-through was discarded and VB was place back into the collection tube, the remaining mixture was transferred to the VB and centrifuged for 1 minute, collection tube was discarded with the flow-through, then VB column was transferred to a new 2ml collection tube. Four hundred (400) µl of W1 buffer was added to the VB column and centrifuged at 16,000g for 30 seconds, flow-through was discarded and VB was placed into collection tube, then centrifuged at 16,000g for 3 minutes to dry the column. Dried VB column was placed in a clean 1.5ml microcentrifuge tube, 50 µl of RNase-free water was added to the centre of VB column, mixture was allowed to stand for 3 mınutes, then centrifuged at 16,000g for 1 minute.

### Primer design

Sequence of E6 gene of HPV 16 were retrieved from NCBI, the selected gene sequence were saved in FASTA format, sequence were imported to BioEdıt, sequence were aligned by running ClastalW from the accessory application Menu, conserved regions were selected as template, the specificities were checked using nucleotide Blast.

### Polymerase Chain Reaction

The PCR was conducted using KOD-FX Neo(Toyobo, Japan) following manufacturer instructions as follows, each PCR mix of 50µL contained, buffer 25µL, 1.3 µL of forward and reverse primer each, DNP of 10µL, molecular grade water of 10 µL, DNA template 1.4 µL, KOD of 1 µL. the following PCR conditions were used, 95°C for 5 minutes, followed by 35 cycles of 94°C for 30 seconds, 60°C for 30 seconds, 72°C for 30 seconds, final extension at 72°C for 5 minutes, then held for 16°C.

### Molecular docking

#### Hardware and software

All computational simulation studies were performed using (Intel Core i5-2430M) 6.00GB RAM with processor 2.40 GHz on Windows 7 operating system. Docking analysis was carried out using bioinformatics software including PyRx virtual screening software (AutoDock Vina) and the visualization of structure was carried out using Pymol molecular graphic system. Online resources were also used in this study.

### Docking Procedure

#### Preparation and Optimization of Structures

##### Ligand Structures

For this present study, ligands were downloaded from zinc12 (zinc.org), and saved in mol2 format, they were converted into pdbqt format by autodock vina.

Preparation and Optimization of L1 Protein Structure Pentamer structure of L1 protein of human papillomavirus (PDB Code:2r5h) was retrieved from the protein data bank (www.rcsb.org/pdb). This was done by typing the PDB code, 2r5h on the protein data bank search engine. Furthermore, the receptor was saved in a receptor file folder, by selecting 2r5h→ download files → PDB format→ download. After saving, the file was opened by double clicking the folder containing the receptor structure which comes out in Pymol. The receptor was prepared by deleting protein and water via edit then delete.

#### Receptor-Ligand docking using PyRx virtual screening software

AutoDock Vina was used to carry out the protein-ligand docking15. Both the ligands and receptor (2r5h) were converted from pdb files to pdbqt (protein data bank, partial charge Q and atom type T) files (Vina input file format). AutoDock Tools (ADT) was used to prepare L1 protein of the human papillomavirus by the complete addition of hydrogen atoms to the receptor's carbon atoms. Non-polar hydrogen were added to the docked ligands. For Lamarckian genetic algorithm, a maximum number of 15 × 105 energy evaluations, 27,000 maximum generations, 0.02 gene mutation rate and 0.8 crossover rate were used16. Each ligands had two hundred independent docking runs.

#### Conversion of PDB/Mol2 files into PDBQT files

**Figure 1:**
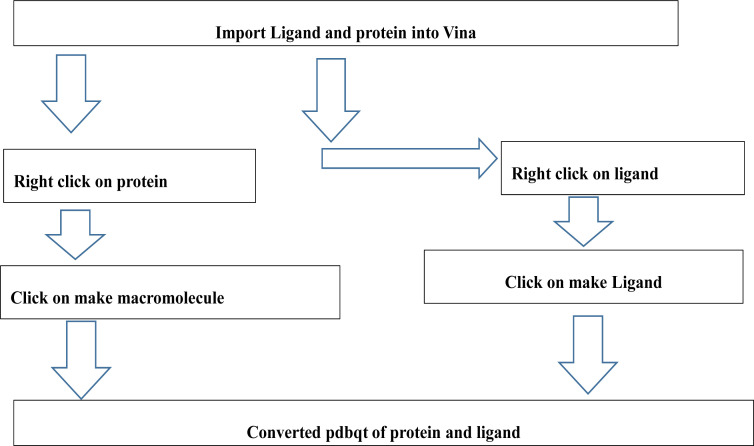
conversion of PDB/Mol2 files to PDBQT files

AutoDock Vina cannot recognise any file format except a pdbqt files format, therefore ligands and protein have to be converted from the downloaded file format to pdbqt format following the procedure as shown in Figure 1.

#### Calculations of binding affinity procedure with autodock vina of PyRx software

**Figure 2:**
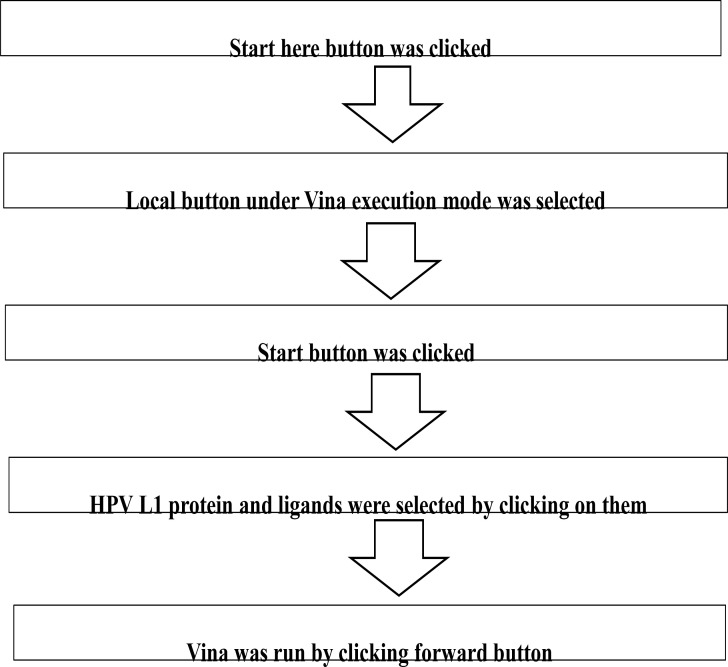
Calculations of binding affinity procedure with autodock vina of PyRx software

Calculation of the binding affinity was done as shown in Figure 2. The results of the calculations were displayed in tabular form with the Binding Affinity (kcal/mol) values. The More negative the binding affinity, the better the orientation of the ligand in the binding site of 2r5h. Furthermore, all the docked calculation results were saved in a location specified as workspace by clicking on File→ Export → Saved. This procedure was applied to all other ligands in each of the data sets and 2r5h to calculate their binding affinity (binding or docking scores).

#### Identification of binding site and hydrophobic interaction between ligand(s) and receptor

The pymol was opened by double clicking its icon on the desktop of a laptop computer to lunch its work space. From the file menu, Open was clicked followed by macromolecule folder containing the docked results, all supported format was changed to all files, receptor was clicked followed by control + ligand. By clicking open ligand and receptor were exported to pymol.

The ligand was selected, copied and pasted on the page containing the receptor.

From the right upper tools 2r5h was selected.

Action was right clicked Find→Polar contact→Within selection shown in figure.

#### Determination of bond length in Angstrom

Wizard was clicked → mearsurement → Show distance.

### Statistical analysis

Statistics analyses were performed using Chi-square with aid of an SPSS version 16.0, and data were presented in tables and figures.

## Results

The low grade squamous intraepithelial lesion (LGSIL) in this study was 21, with about 57% of the low-grade lesion been positive for HPV type 16 virus, none of the participants in this study has carcinoma in situ, only 6 subjects showed cytology of high grade squamous intraepithelial lesion (HGSIL) with all been positive for HPV type 16. The P value is statistically significant (<0.05).

HPV type 16 E6 gene sets of oligonucleotides were used in amplifying HPV DNA using PCR, the PCR products were visualized by 1% agarose gel electrophoresis with Ethidium bromide. As shown in Table 2, 16.9% of the 144 participants were positive for HPV type 16, indicating presence of tumour gene while 83.1% were negative.

**Table 2:** PCR result

Result	Frequency	Percentage (%)
Positive	24	16.9
Negative	120	83.1
Total	144	100.0

Molecular docking studies were carried out between the targets (pentamer structure of major capsid protein L1 of HPV type 16; 2r5h) which has 3 chains, and its inhibitors, active components of Echinacea (chicoric acid, echinacoside, curcumin) The best three docking scores is seen on compounds 2r5h_zinc_33737268, 2r5h_zinc_95098864 and 2r5h_zinc_899824 due to their low binding affinity (-8.7, -8.6 and -6.8) kcal/mol as depicted in Table 3.

**Table 3:** Selected results of 200 independent run for the active components of Echinacea (chicoric acid, curcumin and Echinacoside) with protein 2r5h (HPV's L1 protein)

Ligand	Binding Affinity	rmsd/ub	rmsd/lb
2r5h_zinc_33737268	-8.7	0	0
2r5h_zinc_33737268	-8.6	6.992	3.943
2r5h_zinc_33737268	-8.5	2.185	1.358
2r5h_zinc_33737268	-8.4	2.372	1.606
2r5h_zinc_33737268	-8.4	3.345	2.029
2r5h_zinc_33737268	-8.3	8.924	4.438
2r5h_zinc_899824	-6.6	9.031	3.149
2r5h_zinc_899824	-6.5	8.764	2.993
2r5h_zinc_899824	-6.4	6.335	2.528
2r5h_zinc_899824	-6.3	5.651	2.585
2r5h_zinc_95098864	-8.6	0	0
2r5h_zinc_95098864	-8.3	8.723	3.508
2r5h_zinc_95098864	-8.3	7.656	3.725
2r5h_zinc_95098864	-8.2	3.277	2.392

## Discussion

HPV type 16 has been the major cause of cervical lesion in Africa, it causes 49% of cervical cancer in the continent, higher than any other HPV serotype. HPV type 16 is responsible for 70 percent of all cervical cancer alongside with type 1817. Of the 144 participants recruited in this study 16.9% were positive for HPV type 16, which is slightly higher than the study carried out in Kano, Nigeria where they reported prevalence of 15.8% for HPV type 16, though their study recruited fewer subjects (50) than this study^18^. A study carried out in Lagos, Nigeria, reported the prevalence of HPV type 16 to be 46.9%, which is not in accordance with the present study^19^. Another study reported 23.5%^20^, while another study carried out in south western Nigeria reports an HPV 16 prevalence of 3.5% which is very low compared to that in this study^21^.

This study shows there is an association between infection with HPV 16 and cervical lesions, this is consistent with a study where the strength of the association was even demonstrated with odds ratio of 182, showing a strong association between HPV 16 and cervical lesions^22^. As well as another study which showed HPV 16 is a strong risk factor for cervical lesion^20^.

The population awareness about HPV infections and its vaccine in Africa is low. The cost per person on HPV vaccination is beyond what an average citizen can afford, Herbal therapy such as Echinacea has been reported to not only minimize viral replication but also improve the immune system, with an easy accessibility and affordability prospects in Nigeria^23^. Even though there is currently no scientific rationale for the efficacy of Echinacea therapy against HPV infections^23^, this paper provides basis for future researches, to provide scientific explanation on the plants potentiality for the treatments of HPV infection.

Molecular docking studies were carried out between the targets (pentamer structure of major capsid protein L1 of HPV type 16) which has 3 chains, and its inhibitors (chicoric acid, echinacoside, and curcumin) to fulfil most of the requirements to be used as a therapeutic agent. One of the criteria for a successful therapeutic agent to be fulfilled is the binding energy, binding affinity depends on thform and quantity of bond between target protein (2r5h) and the ligands (chicoric acid, echinacoside, and curcumin), the lower the binding energy, the stronger the relationships would be and the better the binding affinity. Also Root Mean Square Deviation (RMSD) values is primarily used to evaluate protein stability and predict protein conformational changes, ideally RMSD of less than 1.5 Å shows the best protein stability. In this study only RMSD of less than 1.5 Å was considered. All the compounds were found to inhibit strongly by completely occupying the active sites in the target protein (2r5h). In this study the inhibitors occupied the AA 50–60 of the L1 protein, which is situated in the DE-Loop of the HPV L1 protein. Most of the ligands were found to be having polar contact with the receptor (2r5h). The best two docking results were zinc_33737268 (chicoric acid) with a binding energy of -8.7 kcal/mol which is lower than the binding energy reported for Withaferin A docked against HPV E6 protein in a study by Kumar et al. (2014), though in the same study carrageenan showed a much lower binding energy when docked against E6 protein, but the present study targeted the L1 protein which is a more conserved region, the L1 also has the heparin binding site in its loop, which plays important role in high risk HPV infection^24^. However many HSPG-mimicking compound have failed clinical phase III trials, which is explained to be due to dilution of this antiviral agent by body fluid, probably leading to loss of infectious particle^25^. The chicoric acid shows polar interactions with Ala139, Leu126, Asp127, Lys 125, Asn 124, Arg 144, Ala 134, Tyr355, Lys 356, and Gly256 of the target protein. With bond length (1.20 Å, 1.52 Å). Ligand zinc_95098864 with binding score of -8.6 kcal/mol forms bonds (2.50 Å and 3.70 Å) with Lys 125, Asn 124, Thr 226, Ser 227, Leu 275, Leu 222, Gly 183, Cys 185, Ala 264 and Pro 182. Therefore, Chicoric acid components of Echinacea purpurae plant may have anti-L1 protein bioactivities.

## Conclusion

Numerous researches are currently ongoing in order to identify promising therapeutic agents for the management of HPV associated diseases, advancement in molecular modelling and bioinformatics are very important in validating those therapeutic agents using in silico analysis. Although, few researchers have worked on the inhibitory action of natural compounds against L1 protein. The L1 is a conserved region and its loops also have a heparin binding site that plays an important role in high risk HPV infection. This study provides promising data on how the plant Echinacea pupurae can be used to manage HPV-related infections by inhibiting the action of L1 protein when confirmed in the wet laboratory.

## Figures and Tables

**Figure 3:**
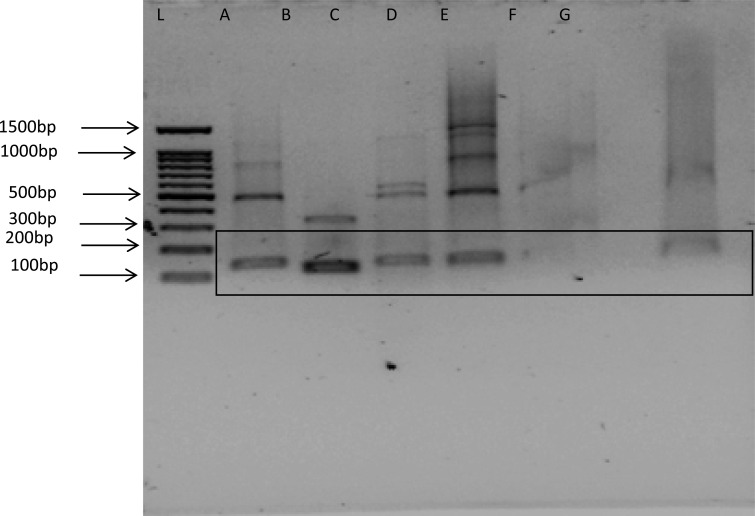
Agarose gel showing polymerase chain reaction on amplified product of HPV 16 E6 gene. L-100 base pair (bp) DNA ladder, A-positive control (band size=119), B, C and D- positive for HPV, E, F, G- negative for HPV

**Figure 4:**
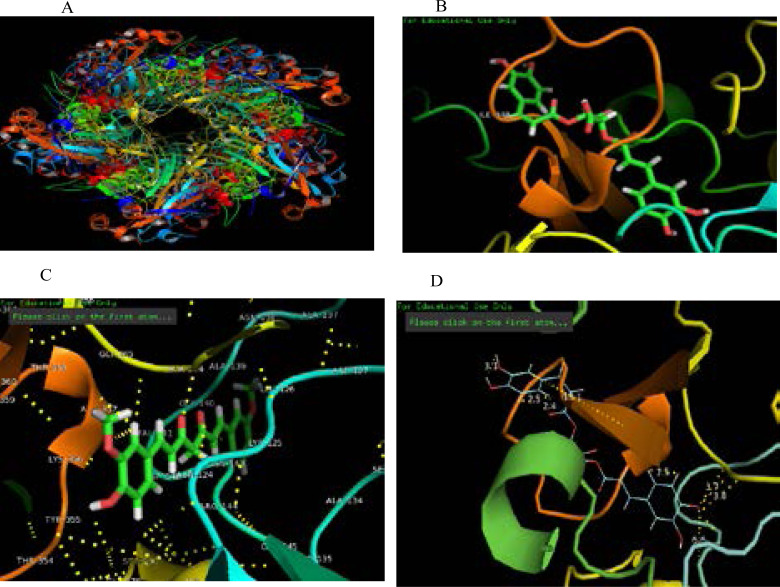
Crystal structure of HPV 16 L1 pentamer and Ligands A: Cartoon structure of HPV 16 L1 pentamer (2R5H), B: Ligand chicoric acid in receptor pocket, C: Ligand echinacoside in receptor pocket showing amino acid residues, D: Ligand chicoric acid docked against pentamer structure of major capsid protein L1 of HPV showing polar contact and bond length.

**Table 1:** Distribution of HPV 16 according to cytology and clinical symptoms

Variables	Values	HPV 16 positive	HPV 16 Negative	P value
Cytology	Negative	6	111	**<0.05**
	LGSIL	12	9	
	HGSIL	6	0	
	Carcinoma in situ	0	0	
